# The increased concentration of macrophage migration inhibitory factor in serum and cerebrospinal fluid of patients with tick-borne encephalitis

**DOI:** 10.1186/s12974-017-0898-2

**Published:** 2017-06-24

**Authors:** Sambor Grygorczuk, Miłosz Parczewski, Renata Świerzbińska, Piotr Czupryna, Anna Moniuszko, Justyna Dunaj, Maciej Kondrusik, Sławomir Pancewicz

**Affiliations:** 1 Department of the Infectious Diseases and Neuroinfections, Medical University in Białystok, ul. Żurawia 14, 15-540 Białystok, Poland; 2 Department of Infectious Diseases and Hepatology, Pomeranian Medical University in Szczecin, ul. Arkońska 4, 71-455 Szczecin, Poland

**Keywords:** Tick-borne encephalitis, Innate immunity, Blood/cerebrospinal fluid barrier, Macrophage migration inhibitory factor, Tumor necrosis factor alpha, Toll-like receptor 3

## Abstract

**Background:**

Host factors determining the clinical presentation of tick-borne encephalitis (TBE) are not fully elucidated. The peripheral inflammatory response to TBE virus is hypothesized to facilitate its entry into central nervous system by disrupting the blood-brain barrier with the involvement of a signaling route including Toll-like receptor 3 (TLR3) and pro-inflammatory cytokines macrophage migration inhibitory factor (MIF), tumor necrosis factor-α (TNFα), and interleukin-1 beta (IL-1β).

**Methods:**

Concentrations of MIF, TNFα, and IL-1β were measured with commercial ELISA in serum and cerebrospinal fluid (CSF) from 36 hospitalized TBE patients, 7 patients with non-TBE meningitis, and 6 controls. The CSF albumin quotient (AQ) was used as a marker of blood-brain barrier permeability. Single nucleotide polymorphisms rs3775291, rs5743305 (associated with TLR3 expression), and rs755622 (associated with MIF expression) were assessed in blood samples from 108 TBE patients and 72 non-TBE controls. The data were analyzed with non-parametric tests, and *p* < 0.05 was considered significant.

**Results:**

The median serum and CSF concentrations of MIF and IL-1β were significantly increased in TBE group compared to controls. MIF concentration in serum tended to correlate with AQ in TBE, but not in non-TBE meningitis. The serum concentration of TNFα was increased in TBE patients bearing a high-expression *TLR3* rs5743305 TT genotype, which also associated with the increased risk of TBE. The low-expression rs3775291 *TLR3* genotype TT associated with a prolonged increase of CSF protein concentration. The high-expression *MIF* rs755622 genotype CC tended to correlate with an increased risk of TBE, and within TBE group, it was associated with a mild presentation.

**Conclusions:**

The results point to the signaling route involving TLR3, MIF, and TNFα being active in TBE virus infection and contributing to the risk of an overt neuroinvasive disease. The same factors may play a protective role intrathecally contributing to the milder course of neuroinfection. This suggests that the individual variability of the risk and clinical presentation of TBE might be traced to the variable peripheral and intrathecal expression of the mediators of the inflammatory response, which in turn associates with the host genetic background.

## Background

Tick-borne encephalitis (TBE) is a zoonotic disease caused by TBE virus (TBEV) belonging to genus *Flavivirus*, family *Flaviviridae*, transmitted by *Ixodes* ticks and endemic in the area stretching from Central Europe to Eastern Asia. An infection with Western (European) TBEV subtype may be asymptomatic and result in a mild flu-like episode or in a biphasic disease involving the central nervous system (CNS) in the second phase, when it presents as meningitis, meningoencephalitis of variable severity, or menigoencephalomyelitis [1–6]. The outcome may be decided at several stages of the infection, including the immune/inflammatory response at the tick bite site and during a systemic infection, the penetration across the blood-brain barrier (BBB), and finally the response within CNS [7, 8]. Especially, the inflammation and innate immunity in the periphery may be protective in the early stage of the infection but may also contribute to TBEV spread and facilitate CNS entry [8]. In a study by Palus et al., the mortality rate and virus titers in blood and brain tissue differed drastically between the sensitive and resistant mice strains when infected subcutaneously, but the differences were much reduced in intracranial infection, suggesting a critical role of the peripheral response and/or the control of the TBEV entry into CNS in determining the outcome [9]. The influence of the genetic background on the sensitivity to TBEV in laboratory animals has an analog in the naturally observed variability of the clinical presentation of human TBEV infection. Several single nucleotide polymorphisms (SNPs) in genes related to inflammation and immune response, including interferon and chemokine signaling, associate with the susceptibility to neuroinvasive TBEV infection and its presentation in humans [10–12]. As a contribution from each of the identified factors is relatively small, the significant role of additional still undefined polymorphisms is likely.

Macrophage migration inhibitory factor (MIF) is a pro-inflammatory cytokine pivotal in initiating inflammatory responses [13]. It is synthesized constitutively and released under pathogen stimulation by T lymphocytes, other leukocytes, and by other cell types, including epithelial cells [13, 14]. Its effect is anti-apoptotic, pro-inflammatory, promoting Th1-type response, and through T cell proliferation and IL-2 expression contributes to the initiation of the adaptive immunity [13]. MIF is located at the onset of the inflammatory cascade upstream of TNFα and acts through effector cytokines, including TNFα, INF-γ, IL-1β, IL-2, IL-6, and IL-8, being up-regulated by TNF-α and INF-γ in a positive feedback loop [13, 15, 16]. The MIF expression is individually variable in association with common genetic polymorphisms in *MIF* gene, including an SNP -173 G/C (rs7555622) and a microsatellite repeat sequence -794 CATT5-8 (rs5844572), which influences the clinical course of infectious diseases and risk of autoimmunity [17–19]. The variable expression of MIF has been shown to correlate with the clinical course of infectious diseases, suggesting its involvement in response to pneumonia and viral meningitis, but also detrimental effects of its over-expression in purulent meningitis and dengue [20–25]. Studies on animal models suggest an immunopathogenic role of MIF in flavivirus encephalitis [26, 27]. In a study by Arjona et al., blocking MIF activity improved the survival of West Nile virus (WNV)-infected mice, reducing the systemic and intrathecal synthesis of pro-inflammatory cytokines (TNFα, IL-6, IL-12), the BBB permeability, the CNS viral burden, and inflammatory infiltrate [26]. As these effects are absent when WNV is injected directly into the brain, the detrimental effect of MIF probably depends on the peripheral inflammatory response. The mechanism suggested by the authors is a BBB disruption by pro-inflammatory mediators up-regulated in the course of a systemic inflammation, facilitating virus entry into the CNS [26]. The authors have also confirmed an incresed expression of MIF in patients with WNV encephalitis [26].

Toll-like receptor 3 (TLR3) is a pattern recognition receptor binding double-stranded RNA and involved in anti-viral defense [28, 29]. TLR3 activation results in the synthesis of pro-inflammatory cytokines (including TNFα, IL-6, and chemokines) as well as type I and III interferons [28, 30]. Several SNPs in *TLR3* gene associated with a variable TLR3 activity have been described [29, 31, 32]. Of these, rs3775291 is a common variant associated with the TLR3 ligand-mediated signaling reduction by about 30% [29]. In mice lacking TLR3, the WNV infection is characterized not only by a weaker inflammatory response, lower cytokine expression, and higher viral load in periphery but also by a reduced virus penetration into and milder inflammatory infiltrate within CNS [33]. The response to WNV injected directly into the brain is not altered, which mimics the results of the impaired MIF function and suggests that TLR3 and MIF may belong to the same signaling pathway involved in BBB disruption and facilitating WNV entry into CNS [26, 33]. The effect described by Wang et al. depends on the presence of active TNFα receptor 1 [33]. Importantly in that context, TNFα is up-regulated by MIF in West Nile virus infection in mice [26] and has been shown to increase BBB permeability, acting synergistically with another pro-inflammatory cytokine, interleukin 1 beta (IL-1β) [34–36].

To our knowledge, the phenotypic expression of MIF and TLR3 have not been evaluated in human flavivirus encephalitis other than caused by WNV and their role in TBE pathogenesis is especially unclear. A study on TBEV-infected mice suggests that this virus causes BBB disruption only after the onset of encephalitis, too late to promote CNS penetration like in the WNV encephalitis model [37]. On the other hand, the results of human genetic association studies suggest that the homozygocity for the low-expression rs3775291 allele of *TLR3* is protective against neurotropic TBEV infection, consistent with the idea of TLR3 contributing to TBEV entry into CNS [38–40].

We have attempted to assess if a pathway involving TLR3, MIF, and TNFα may contribute to the pathogenesis of TBE in humans in an analogous way as in the animal WNV encephalitis models. We have studied a group of hospitalized TBE patients, measuring concentration of MIF, TNFα, and IL-1β in serum and CSF. We have also genotyped two SNPs associated with TLR3 expression, rs3775291 and rs5743305, as well as an SNP associated with MIF expression, rs755622, in a larger group of TBE patients and controls from the same area and analyzed correlations of the studied parameters with the TBE clinical presentation and with CSF inflammatory parameters, especially the albumin concentration as the measure of the BBB disruption.

## Methods

### Patients

The study group consisted of 36 patients with a TBEV CNS infection, hospitalized in the Department of the Infectious Diseases and Neuroinfections of the Medical University in Białystok in 2013–2014. The diagnosis was based on the history of a recent tick bite or exposition to ticks in TBE endemic areas, clinical symptoms (acute febrile illness with a prominent headache and meningeal signs), CSF pleocytosis, and specific anti-TBEV IgM antibodies detection in at least one serum or CSF sample. Thirteen patients had uncomplicated meningitis (M), 21 were diagnosed with meningoencephalitis (ME) based on an altered mental status and/or focal neurologic deficits, and two presented with additional brachial paresis and were diagnosed with meningoencephalomyelitis (MEM). Of the ME patients, eight had mild presentation with isolated focal neurological symptoms only (ataxia, tremor, fasciculations, paresthesia), nine with moderately severe presentation had altered mental status (lethargy, apathy, impaired verbal contact) typically accompanied by focal neurologic deficits (including mild focal pareses in three), and four with severe encephalitis presented with a disorientation and/or loss of consciousness. One of MEM patients had transient brachial paresis and a mild cognitive dysfunction (classified as a moderately severe infection) while the other suffered a severe disease with a loss of consciousness and multifocal pareses. Additionally, seven patients with lymphocytic meningitis of non-TBEV etiology (no history of tick bites, negative TBEV serology) were studied. Six of them had mild meningitis and were hospitalized during an Echovirus 30 meningitis outbreak, with an epidemiologic history of a recent contact with a confirmed or suspected Echovirus infection case. One patient presented with a meningoencephalitis of unknown etiology with tremor and oculomotor dysfunction. The patient data are presented in Table 1. The healthy control group consisted of six blood donors. The non-inflammatory control CSF samples (*n* = 6) were obtained from patients who were hospitalized with the suspicion of meningitis but in whom neuroinfection was eventually excluded and no abnormalities in the CSF general examination were detected.

**Table 1:** The patients included in the cytokine study group

	Sex	Age	Etiology	Presentation	Altered consciousness	Neurologic symptoms	CRP (mg/l)	Leuko-cytosis^a^	CSF on admission				
									Pleocytosis^a^	Neutrophils^a^	Lymphocytes^a^	Protein^b^	Albumin^b^
1	F	32	TBE	M	(−)	(−)	8.2	11,030	174	14	146	70	50
2	F	31	TBE	M	(−)	(−)		6720	69	1	66	51	35
3	M	46	TBE	M	(−)	(−)	9.7	13,790	40	4	26	53	37
4	M	21	TBE	M	(−)	(−)	5.6	13,000	228	23	160	81	58
5	M	50	TBE	M	(−)	(−)	12.6	8790	146	39	69	53	36
6	F	70	TBE	M	(−)	(−)	0.8	6140	30	4	23	42	29
7	M	65	TBE	M	(−)	(−)	7.5	8680	46	8	30	85	60
8	F	18	TBE	M	(−)	(−)	2	13,210	266	59	200	50	38
9	M	35	TBE	M	(−)	(−)	1.4	13,440	85	5	75	40	28
10	M	24	TBE	M	(−)	(−)	6.1	7330	76	10	57	61	48
11	M	30	TBE	M	(−)	(−)	22	13,210	122	21	71	64	49
12	M	44	TBE	M	(−)	(−)	5.9	13,960	51	9	37	43	30
13	M	44	TBE	M	(−)	(−)	9.1	11,140	25	10	16	46	35
14	F	49	TBE	ME	(−)	Cerebellar	32.8	10,180	75	22	50	48	40
15	M	18	TBE	ME	(−)	Double vision, unsteady gait	16.1	11,730	103	50	35	59	39
16	F	43	TBE	ME	(−)	Cerebellar	10.8	11,900	56	17	34	45	29
17	M	28	TBE	ME	(−)	Fasciculations, tremor	10.6	13,900	258	21	224	101	73
18	M	28	TBE	ME	(−)	Cerebellar	2.6	9680	176	128	11	48	34
19	M	50	TBE	ME	(−)	Cerebellar	3.1	11,790	134	7	122	65	46
20	M	60	TBE	ME	(−)	Cerebellar	7.4	9150	8	3		38	24
21	F	55	TBE	ME	(−)	Cerebellar	6.2	11,270	64	64	0	66	49
22	M	50	TBE	ME	Mild impairment	(−)	17.5	10,120	32	1	24	109	87
23	M	49	TBE	ME	Mild impairment	(−)	10.3	7070	50	6	37	48	37
24	M	27	TBE	ME	Mild impairment	(−)	0.3	10,900	16			46	ND
25	M	51	TBE	ME	Mild impairment	Transient hemiparesis	9.5	15,100	22	7	13	29	ND
26	M	55	TBE	ME	Mild impairment	Dysarthria, asymmetric reflexes		7020	67	5	46	103	86
27	M	65	TBE	ME	Mild impairment	(−)	0.8	10,085	94	11	61	56	40
28	M	44	TBE	ME	Mild impairment	(−)	15.6	17,060	352	74	243	96	29
29	M	60	TBE	ME	Mild impairment	cerebellar, tremor	16.7	9260	88	22	53	82	46
30	M	38	TBE	ME	Mild impairment	(−)	19.3	11,470	239	38	172	106	85
31	F	45	TBE	ME	Deeply disoriented, agitated	VI cranial nerve paresis	0.5	18,450	1268	1192	38	130	94
32	M	65	TBE	ME	Deeply disoriented, agitated	(−)	1.7	4270	4			58	36
33	F	54	TBE	ME	Deeply disoriented, agitated	(−)	17.9	4790	49	22	18	52	36
34	F	45	TBE	ME	Deeply disoriented, agitated	(−)	9.3	11,450	80	10	69	46	36
35	M	57	TBE	MEM	Mild impairment	Paresis of the right arm	16.7	15,100	17	3		82	64
36	F	76	TBE	MEM	unconscious	Paresis of the right arm, neck, III cranial nerve bilaterally	4	8860	122	6	116	65	ND
37	M	21	N	M	(−)	(−)	2.8	7890	156	97	50	51	39
38	M	40	N	M	(−)	(−)	19.1	10,460	242	5	198	34	30
39	F	36	N	M	(−)	(−)	4.3	12,340	144	37	101	63	48
40	M	17	N	M	(−)	(−)	1.4	8200	142	18	111	36	27
41	F	26	N	M	(−)	(−)	18	8580	69	0	65	31	20
42	M	25	N	M	(−)	(−)	24.5	9210	102	11	59	47	36
43	F	38	N	ME	(−)	Tremor, double vision	1.7	10,260	311	0	302	165	103

The basic clinical and laboratory data of the patients included in the tick-borne encephalitis study group (TBE) and of the controls with meningitis of different etiology (N)

*M* meningitis, *ME* meningoencephalitis, *MEM* meningoencephalomyelitis, *CRP* C-reactive protein, *ND* not done

^a^In cells/μl

^b^In mg/100 ml

The TBE blood and CSF samples were obtained twice: on admission to hospital early in the neurologic phase of the disease (examination I) and before the discharge 10–14 days later (examination II), together with the material drawn for clinically indicated basic laboratory and serologic examinations. The cytokine concentrations were studied in the examination I serum in the whole study group. In five TBE patients (three with meningitis and two with meningoencephalitis), the concentrations were measured in examination II serum and in both CSF samples as well, to assess the dynamics of the cytokine expression and concentration gradients across BBB. In non-TBE meningitis patients, cytokine expression was evaluated in admission serum and CSF, with the exception of one patient in whom CSF sample was not available.

The genotyping was performed in a group of 108 TBE patients hospitalized from 2013 to 2016, including the TBE patients participating in the cytokine expression study. In this group, 46 patients were diagnosed with M, 57 with ME, and 5 with MEM. The control genotyping group consisted of 72 subjects coming from the same study area, including 16 healthy blood donors and 56 hospitalized patients in whom TBE was originally suspected but eventually excluded based on clinical presentation, CSF examination, and negative serology (patients diagnosed with other febrile infections or headache of non-infectious etiology).

### Laboratory techniques

Peripheral leukocytosis, C-reactive protein concentration, serum albumin concentration, and general CSF parameters (total pleocytosis, neutrophil and lymphocyte count, total protein and albumin concentration) were studied with standard laboratory techniques.

Anti-TBEV IgM antibodies were detected with Enzygnost Anti-TBE/FSME IgM kit from Siemens (Munich, Germany), following the standard procedure.

Concentrations of cytokines were measured with commercial Quantikine ELISA kits from R&D Systems (Minneapolis, USA), strictly following the manufacturer’s instructions. The minimal detectable concentration according to the manufacturer’s data was 160 pg/ml for MIF, 1.6 pg/ml for TNFα, and 1.0 pg/ml for IL-1β.

QIAamp DNA Blood Mini Kit (QIAgen, Hilden, Germany) was used to extract genomic DNA from the blood samples collected to EDTA tubes, following the manufacturer’s protocol. DNA was re-suspended in 200 μl of AE buffer (QIAgen, Hilden, Germany) and stored at 4 °C for further analyses. The SNPs rs3775291 and rs5743305 in *TLR3* as well as rs755622 in *MIF* were analyzed using TaqMan SNP (Life Technologies, USA) genotyping assay, based on real-time PCR technology on the StepOne thermal cycler (Applied Biosystems/Life Technologies, Foster City, CA), according to the manufacturer’s protocol. Genotypes were called using TaqMan Genotyper Software v1.0.1 (Applied Biosystems/Life Technologies, Foster City, CA), calculation of Hardy-Weinberg equilibrium (WHE) for each analyzed set of genotypes was performed by this software. No deviation from the WHE was detected for any of the analyzed SNPs.

### Statistical analysis

The data were analyzed with Statistica 10 software (Statsoft, Poland) with non-parametric tests, and *p* < 0.05 was considered significant. Because of their small cohort size, the MEM patients were joined with the ME group into one group of patients with clinically manifesting CNS pathology in some analyses.

Album quotient (AQ) was calculated as CSF albumin/serum albumin ratio and applied as a marker of BBB disruption on admission.

The distribution of genotypes between the TBE and control groups was assessed with a chi-square test.

## Results

### Basic laboratory parameters

**Fig. 1:**
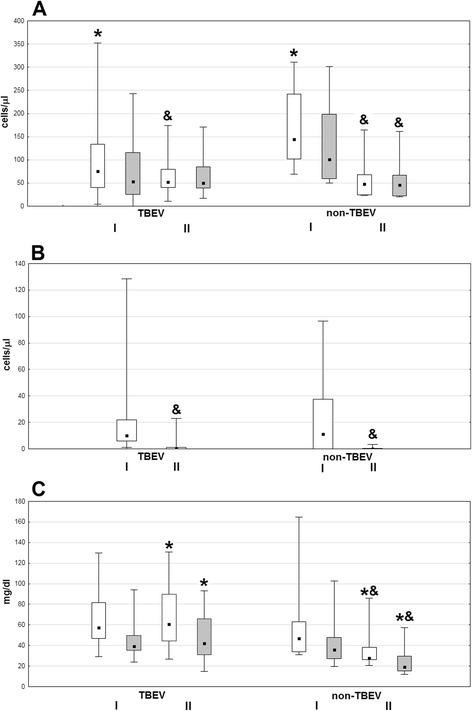
The basic inflammatory parameters of cerebrospinal fluid. The inflammatory parameters of cerebrospinal fluid (CSF) in patients with tick-borne encephalitis virus (TBEV) infection (*n* = 36) and with non-TBEV meningitis (*n* = 7). *I* on admission, *II* follow-up after 10–14 days. **a** Total pleocytosis (*empty boxes*) and lymphocyte count (*filled boxes*). **b** Neutrophil count. **c** Total protein (*empty boxes*) and albumin (*filled boxes*). Shown median (*square*), quartiles (*box*), and minimum/maximum values (*whiskers*). *significant difference between TBEV and non-TBEV infection (*p* < 0.05); & significant decrease in examination II in comparison with examination I (*p* < 0.05)

On admission, median pleocytosis was lower in TBE than in non-TBE meningitis, with a relatively high neutrophil fraction, while CSF protein and albumin concentration and AQ did not differ significantly. The improvement of CSF parameters in the TBE group was limited at the time of examination II: median pleocytosis fell by about 30% and lymphocyte count, protein and albumin concentration, and AQ did not change, while all these parameters decreased evidently in non-TBE-meningitis patients. As a result, examination II pleocytosis was similar in both groups while the protein and albumin concentrations and AQ were over twofold and significantly (*p* < 0.05) higher in TBE (Fig. 1).

**Fig. 2:**
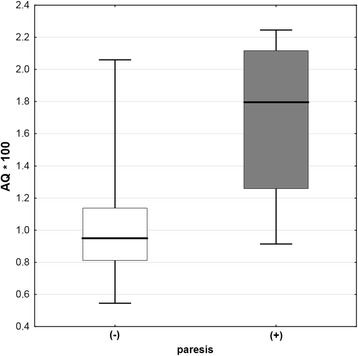
The cerebrospinal fluid albumin quotient dependent on the presence of paresis. The albumin quotient (AQ) in cerebrospinal fluid of patients with tick-borne encephalitis (TBE) on admission to hospital is significantly higher in patients presenting with paresis (*n* = 6) than in those without paresis (*n* = 30) (*p* < 0.05)

There were no significant differences in the blood and CSF inflammatory parameters between the M and ME/MEM groups of the TBE patients or between the patients with and without altered consciousness on admission. Patients presenting with paresis had significantly higher CSF AQ and albumin concentration on admission compared to the rest of the TBE group (*p* < 0.05) (Fig. 2).

### Cytokine concentrations

**Fig. 3:**
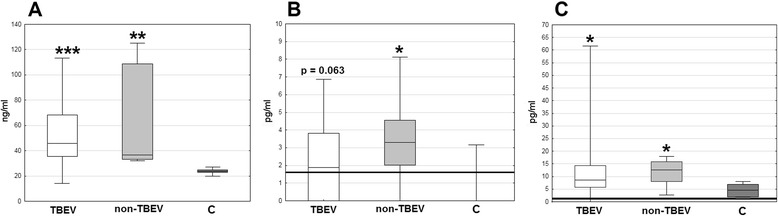
The concentrations of MIF, TNF-α, and IL-1β in serum on admission to hospital. The concentration of MIF (**a**), TNF-α (**b**), and IL-1β (**c**) in serum on admission to hospital in patients with tick-borne encephalitis (TBE—*empty boxes*; *n* = 36), other lymphocytic meningitis (non-TBE—*light-filled boxes*; *n* = 7) and controls (**c** *dark-filled boxes*; *n* = 6). Shown are median (*horizontal line*), quartiles (*box*), and minimum/maximum values (*whiskers*). The *thick horizontal line* at the bottom denotes the lower detection limit in **b** and **c**. ***significant difference in comparison with controls (*p* < 0.001); **significant difference in comparison with controls (*p* < 0.01); *significant difference in comparison with controls (*p* < 0.05); the difference between the TBE group and controls not reaching the level of the statistical significance is shown directly in **b**

On admission, the median MIF concentration was significantly elevated in serum in both TBE (*p* < 0.001) and non-TBE patients (*p* < 0.01) (Fig. 3a). TNFα was detectable and tended to be elevated in most of the meningitis/encephalitis serum samples, significantly in the non-TBE group (*p* < 0.05) but the increase in comparison with controls in the TBE group was not significant (*p* = 0.063) (Fig. 3b). IL-1β serum concentration was significantly elevated both in TBE and non-TBE meningitis (*p* < 0.05) (Fig. 3c). There was no significant difference between cytokine concentrations in the TBE and non-TBE groups. There was also no correlation of the concentrations of the cytokines with one another.

**Fig. 4:**
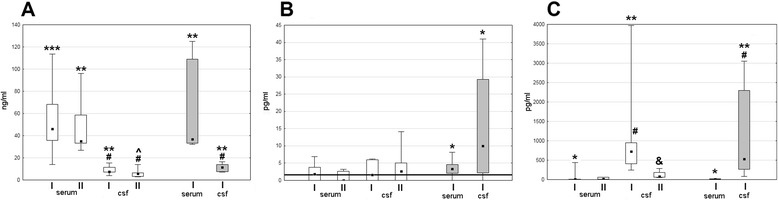
The concentrations of MIF, TNF-α, and IL-1β in serum and cerebrospinal fluid in the acute and convalescent phase of TBE. Comparison of the concentration of MIF (**a**), TNF-α (**b**), and IL-1β (**c**) in serum and cerebrospinal fluid (CSF) obtained on admission (I) and during follow-up examinations before the discharge from hospital 10–14 days later (II). *Empty boxes*—TBE patients (*n* = 5), *filled boxes*—patients with non-TBE aseptic meningitis (*n* = 6). Shown are median (*square*), quartiles (*box*), and minimum/maximum values (*whiskers*). The *thick horizontal line* at the bottom denotes the lower detection limit in **b**. ***value significantly increased in comparison with healthy controls (*p* < 0.001); **value significantly increased in comparison with healthy controls (*p* < 0.01); *value significantly increased in comparison with healthy controls (*p* < 0.05); ^*p* = 0.052 for comparison with healthy controls; & significant decrease on follow-up in comparison with admission (*p* < 0.05); ^#^significant difference in CSF in comparison with a simultaneous concentration in serum (*p* < 0.05)

In patients in whom CSF samples were analyzed, MIF concentration was significantly higher (*p* < 0.05) in serum than simultaneously in CSF (where it was still elevated in comparison with controls, *p* < 0.01), and for IL-1β, the gradient was reverse with a several-fold higher CSF concentration (*p* < 0.01). There was no difference of CSF MIF and IL-1β concentrations between TBE and non-TBE meningitis groups. The TNFα concentration in CSF was significantly elevated in non-TBE (*p* < 0.05), but not in TBE patients, analogously to the situation in serum (Fig. 4).

In the follow-up examination, there was a tendency for the decrease of the cytokine concentrations, but the only statistically significant change was a decline of CSF IL-1β (*p* < 0.05) (Fig. 4).

**Fig. 5:**
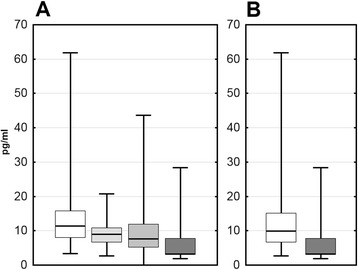
The dependence of IL-1β serum concentration on the clinical severity. Concentration of IL-1β in serum on admission in TBE patients, stratified dependent on clinical severity of the presentation (**a**) and consciousness level (**b**). **a** Patients with uncomplicated meningitis in *white* (*n* = 13) and with neurologic involvement of severity increasing from mild (*n* = 8) through moderate (*n* = 10) to severe (*n* = 5) (as defined in the “Methods” section) in the *darkening gray shades*. **b** Patients with normal mental status (*n* = 21) in *white*, with deep disorientation or loss of consciousness (*n* = 5) in gray. Shown are median (*square*), quartiles (*box*), and minimum/maximum values (*whiskers*). The trend towards lower concentrations in more severely ill patients did not reach a level of statistical significance

The serum cytokine levels did not differ between the groups of TBEV-infected patients defined by the clinical form of the disease, its severity, presence or lack of mental status alterations, and paresis. Only IL-1β showed a consistent but statistically non-significant trend towards lower concentrations in patients with a more severe disease and more severely altered mental status (Fig. 5). In CSF, higher concentration of IL-1β was observed in two patients with severe encephalitis than in three with meningitis, opposite to the tendency in serum. MIF CSF concentrations in those two patients were diverse, the lowest and the highest in the group.

**Fig. 6:**
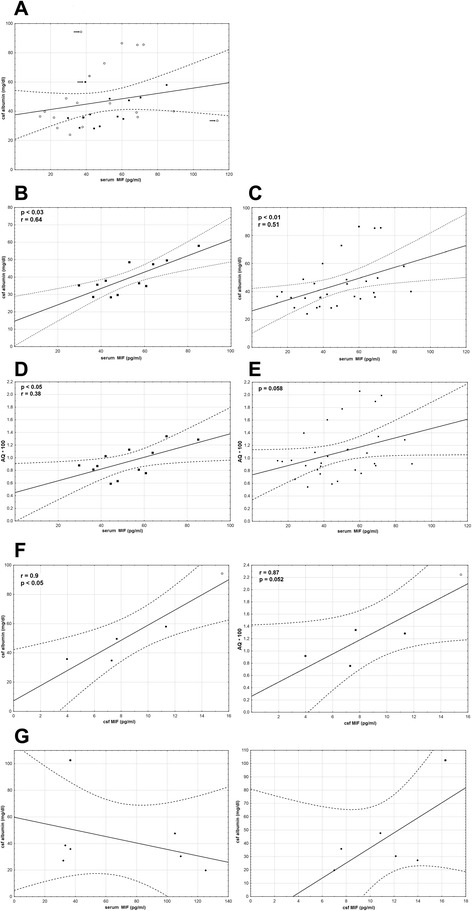
Correlation of the cerebrospinal fluid albumin concentration and albumin quotient with the serum MIF concentration. Correlation between the concentration of albumin in the cerebrospinal fluid (CSF) expressed in mg/dl or of the CSF albumin quotient (AQ) on admission to hospital (*vertical axis*) and the simultaneous concentration of MIF in serum or CSF expressed in pg/ml (*horizontal axis*). **a** Concentration of MIF in serum obtained on admission to hospital from 33 TBE patients and CSF albumin concentration. *Filled squares* denote patients with meningitis (M), *empty circles* patients with meningoencephalitis (ME), a *circle with a cross* in **a**—a patient with meningoencephalomyelitis (MEM). The *arrow* show data points representing one patient with M and two with ME who were excluded from the analyses presented, respectively, in **b**, **c**. **b** The same as in **a**, in a subgroup of meningitis patients, the outlying data from one patient were removed. Positive correlation with *p* < 0.03. **c** The same as in **a**, but the outlying data from one patient with meningitis and two with meningoencephalitis were removed. Positive correlation with *p* < 0.01. **d** The same as **b**, but with AQ*100 instead of the CSF albumin concentration. Positive correlation with *p* < 0.05. **e** The same as **c**, with AQ*100 instead of the CSF albumin concentration. The trend for a positive correlation did not reach the level of statistical significance. **f** Concentration of MIF in CSF on admission in a group of five TBE patients: *left*—a strong positive correlation with CSF albumin concentration (*p* < 0.05), *right*—a tendency for a positive correlation with AQ. *Filled squares* denote patients with meningitis and an *empty circle* a patient with meningoencephalitis as in **a**. **g** Concentration of MIF in six patients with non-TBEV neuroinfection, in serum (*left*) and in CSF (*right*), did not correlate with CSF albumin concentration. *Diamonds*—patients with mild meningitis hospitalized during Echovirus 30 outbreak; *circle*—patient with a moderately severe meningoencephalitis of unknown etiology. The same results were obtained for AQ instead of CSF albumin concentration (not shown)

There was no correlation between serum cytokine concentrations and the cellular parameters of CSF. Serum IL-1β and TNFα did not correlate with CSF protein and albumin concentration and AQ either (not shown). For MIF, there was no correlation with CSF albumin in non-TBE meningitis group and a trend towards positive correlation in TBE, depending mainly on patients with meningitis. In the meningitis subgroup, the correlation became significant with *p* < 0.03 after the exclusion of a single extreme case, and in the whole TBE group with *p* < 0.01 after the exclusion of two outlying patients with ME (Fig. 6a–c). The AQ presented with analogous but weaker trend for a positive correlation, which after exclusion of the same cases was significant (*p* < 0.05) in M subgroup, but did not reach statistical significance in the whole TBE group (Fig. 6d, e).

Cytokine concentrations in CSF tended to correlate positively with the CSF inflammatory parameters, but the low sample numbers hampered the evaluation of this trend. MIF concentration correlated significantly with pleocytosis and lymphocyte count in non-TBE and with CSF protein and albumin in TBE group (*p* < 0.05) while for AQ, the correlation did not reach the statistical significance (*p* = 0.052) (Fig. 6f). In the non-TBE group, the patient with the most severe clinical presentation and unknown etiology had both the highest CSF MIF and albumin concentrations, while for the patients with probable Echovirus meningitis, there was no clear trend (Fig. 6g). TNFα concentration correlated with total pleocytosis, lymphocyte count, and AQ only in non-TBE meningitis (*p* < 0.05). IL-1β correlated with protein and albumin concentration (*p* < 0.05) and tended to correlate positively with AQ in the non-TBE group. It also correlated with CSF neutrophil count in the TBE group, where however, the statistical significance did not hold after removing a single extremely high value (not shown).

### Genotypes

The distribution of the studied *TLR3* and *MIF* genotypes in TBE and control groups is presented in Table 2. The dominant TT genotype in rs5743305 was significantly more common in TBE patients than in the controls (*p* < 0.05), while rs3775291 genotypes were distributed evenly between the groups. In *MIF* rs755622 locus, the high-expression CC genotype was present in three TBE patients and none of the controls, which was not statistically significant.

**Table 2:** The distribution of the *TLR3* and *MIF* genotypes between patients with tick-borne encephalitis and controls

Gene	TLR3						MIF		
SNP	rs3775291			rs5743305			rs755622		
Genotype	CC	TC	TT	TT	AT	AA	GG	GC	CC
TBE (*n* = 108)	57 (53%)	43 (40%)	8 (7%)	46 (43%)*	51 (47%)	11 (10%)	71 (65%)	34 (31%)	3 (3%)
CG (*n* = 72)	36 (50%)	31 (43%)	5 (7%)	19 (26%)*	44 (61%)	9 (8%)	52 (72%)	20 (28%)	0 (0%)

The genotypes of the studied *TLR3* and *MIF* single nucleotide polymorphisms in the group of tick-borne encephalitis patients and in controls with no recorded history of a clinically manifest tick-borne encephalitis virus infection

*TBE* tick-borne encephalitis group, *CG* control group

*Statistically significant difference of the genotype frequency, *p* < 0.05

The distribution of the genotypes in TBE group is shown in Table 3. There was no difference in the frequency of rs3775291 and rs5743305 genotypes between meningitis and ME/MEM patient groups. All three patients with rs755622 CC genotype had uncomplicated meningitis, and the trend for higher frequency of CC in M in comparison with ME/MEM group was significant (*p* < 0.05). The tendency for higher frequency of CG and C allele in M compared to ME/MEM did not reach the level of statistical significance.

**Table 3:** The distribution of the *TLR3* and *MIF* genotypes in patients with tick-borne encephalitis

Gene	TLR3						MIF		
SNP	rs3775291			rs5743305			rs755622		
Genotype	CC	TC	TT	TT	AT	AA	GG	GC	CC
M (*n* = 46)	4 (9%)	14 (30%)	28 (61%)	17 (37%)	24 (52%)	5 (11%)	26 (57%)	17 (37%)	3 (7%)*
ME (*n* = 57)	4 (7%)	26 (46%)	27 (47%)	28 (49%)	24 (42%)	5 (9%)	42 (74%)	15 (26%)	0 (0%)*
MEM (*n* = 5)	0 (0%)	3 (60%)	2 (40%)	1 (20%)	3 (60%)	1 (20%)	3 (60%)	2 (40%)	0 (0%)*

The genotypes of the studied single nucleotide polymorphisms (SNPs) in *TLR3* and *MIF* in the groups of patients with different clinical forms of the tick-borne encephalitis virus infection

*M* meningitis, *ME* meningoencephalitis, *MEM* meningoencephalomyelitis

*Statistically significant difference between M and ME/MEM, *p* < 0.05

**Fig. 7:**
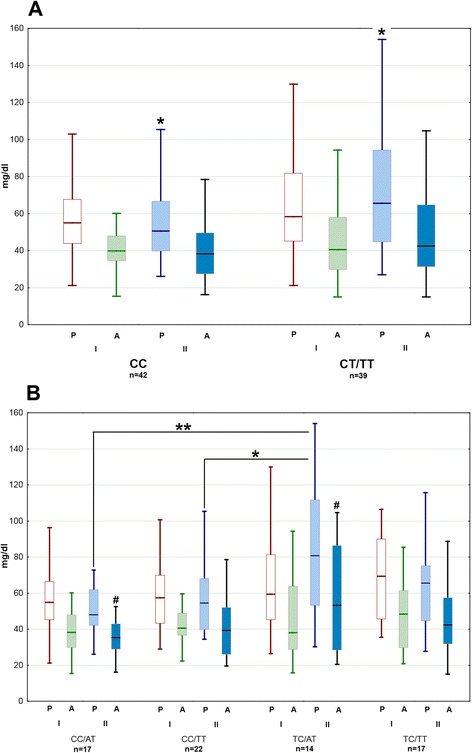
The cerebrospinal fluid albumin and protein concentration dependent on *TLR3* genotypes. Differences of the protein (*P*) and albumin (*A*) concentrations in the cerebrospinal fluid (CSF) obtained on admission (*I*) and during control examinations 12–16 days later (II) between the subgroup of patients stratified according to rs3775291 and rs5743305 genotypes. **a** Comparison between the rs3775291 CC homozygotes and the bearers of the minority T allele (CT and TT genotypes). **b** Comparison between the most frequent rs3775291/rs5743305 genotypes. Shown median (*line*), quartiles (*box*), and minimum/maximum values (*whiskers*). *significant difference between the groups with *p* < 0.05; **significant difference between the groups with *p* < 0.01; ^#^groups differing with *p* = 0.084. Using albumin quotient (AQ) instead of albumin concentration did not reveal additional associations

The peripheral inflammatory parameters (C-reactive protein concentration, leukocytosis) and CSF cellular parameters did not correlate with *TLR3* SNPs (not shown). AQ and CSF albumin concentration did not differ significantly between the genotypes. The CSF protein concentration in examination II was higher in the bearers of T allele and in the CT versus CC genotype in rs3775291 (*p* < 0.05) (Fig. 7a). When comparing the most common combined rs3775291/rs5743305 genotypes, the CSF total protein concentration before discharge was significantly higher in TC/AT than in CC/AT (with *p* < 0.01) and CC/TT (*p* < 0.05) genotype and there was a similar trend for the albumin concentration (*p* = 0.084) (Fig. 7b).

The rs755622 variability did not correlate significantly with the studied laboratory parameters (not shown). However, the two patients with CC genotype in whom the full differential of the admission CSF was available had relatively low lymphocyte count (15 and 19 cells/μl, median in TBE patients 48/μl) with neutrophil predominance.

**Fig. 8:**
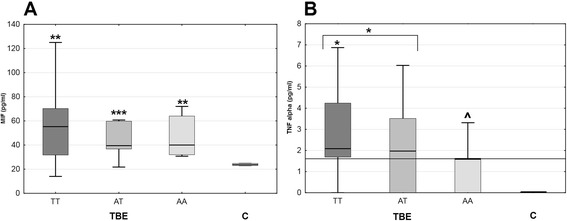
The serum concentrations of MIF and TNFα dependent on *TLR3* genotypes. Concentrations of MIF (**a**) and TNFα (**b**) in serum of TBE patients with TT (*n* = 16), TA (*n* = 14), and AA genotype (*n* = 5) in rs5743305 locus and in healthy controls (*C*, *n* = 6). Shown are median (*line*), quartiles (*box*), and minimum/maximum values (*whiskers*). The *thick horizontal line* denotes the detection limit in **b**. *increased in comparison with controls, *p* < 0.05; **increased in comparison with controls, *p* < 0.01; ***increased in comparison with controls, *p* < 0.001; ^tendency for a lower concentration in AA in comparison with TT/TA with *p* = 0.06

Of the patients with the measured cytokine concentrations, the genotyping data were available in all but one. There were 17 patients with CC genotype, 15 with TC, and one with TT in *TLR3* rs3775291 locus and 16 patients with TT, 14 with TA, and 5 with AA in rs5743305. The presence of A allele (AA or TA genotype versus TT) in rs5743305 tended to associate with lower serum MIF concentration, but the trend was statistically non-significant and in all the genotypes MIF concentration remained up-regulated in comparison with controls. The lack of T allele (AA genotype versus TA and TT) associated with lower TNFα concentration with *p* = 0.06. TNFα was undetectable in two out of five patients with AA genotype and at the detection threshold of 1.6 pg/ml in another two. The patients bearing T allele and with TT genotype had significantly increased TNFα serum concentration in comparison with controls (*p* < 0.05), in contrast with the whole TBE group and with AT and AA genotypes analyzed separately (Fig. 8).

There were no patients with CC genotype in rs755622 *MIF* locus in the cytokine study group, while 20 patients had GG and 15 - GC genotype. The MIF and other studied cytokine concentrations did not differ between these two genotypes.

## Discussion

Data from animal models show that the events occurring during the peripheral virus spread and the penetration through BBB into the CNS may be crucial for the outcome of the neurotropic flavivirus infection [7, 9, 26, 33]. Especially, the BBB disruption caused by a systemic inflammatory response has been hypothesized to determine the WNV entry into the CNS [26, 33], which we have attempted to verify in human TBE in a clinical setting. Patients enrolled in our study had an increased intrathecal total protein concentration and AQ persisting long into the convalescent period, as was described in TBE previously [41]. The prominent increase of AQ points to increased BBB permeability as an important pathogenetic mechanism, but its dynamics is suggestive rather of a protracted BBB impairment during CNS inflammation and healing than to its early disruption preceding neuroinvasion. Similarly, in the mouse TBE model described by Růžek et al., the BBB dysfunction does not occur during systemic infection, but only after the establishment of encephalitis, simultaneously with the up-regulated intrathecal TNFα and IL-6 synthesis [37]. This course of events does not contradict the pathogenetic role of BBB permeability, but postpones it to the later, neurologic phase of the disease. It also implies an alternative mechanism of CNS invasion not dependent directly on BBB disruption by systemic inflammation.

To shed more light on the consequences of BBB dysfunction, we have studied if AQ was related to the clinical presentation and severity of TBE. In WNV infection, the protein concentration in CSF is higher in patients with encephalitis than in those with meningitis [42]. In our previous study, assessing retrospectively the recorded clinical and laboratory data from 687 consecutive TBE patients on admission, we have detected a modestly higher total protein concentration in patients with ME/MEM compared to uncomplicated meningitis; no CSF albumin concentration and AQ were available in most of these patients [1]. This has not been reproduced in a much smaller group included in the current study. However, the admission AQ was higher in patients with paresis than in the rest of the group, consistent with the role of the early BBB impairment in the neuropathology and hinting for a possibility of a specific pathogenesis of particular neurologic complications. We have recently confirmed this observation in another population of 250 TBE patients analyzed retrospectively, in whom CSF albumin on admission was significantly higher in MEM and in patients with spinal paresis (manuscript in preparation).

We have assessed expression of three pro-inflammatory cytokines suspected to mediate increased BBB permeability [26, 33, 35, 36], including MIF which is up-regulated by WNV but according to our knowledge has never been studied in TBE [26]. The main focus was on serum cytokine concentrations early in the course of the disease, which not only represent the early systemic response to TBEV, but might directly contribute to BBB disruption and CNS infection [26, 33]. In a clinical setting, we were not able to study cytokine expression at the most relevant time-point before or during TBEV entry into CNS, as at this stage, patients experience non-specific and usually benign symptoms of peripheral infection and rarely seek medical attention. Instead, we have studied blood samples obtained directly after the admission to hospital, during the early neurologic phase, as soon after the occurrence of neuroinvasion as practically possible. Most of the patients at that stage are febrile and present with a moderate leukocytosis and increased C-reactive protein concentrations [1], which was also true in our study group, suggesting that the inflammatory process in the periphery had not fully resolved yet. In a subgroup of patients, we have additionally studied CSF and convalescent period samples to assess the cytokine concentration gradient and dynamics. Although MIF, TNFα, and IL-1β were expected to influence the BBB function as members of a common signaling route, we found each of them following a different pattern of expression, suggesting that they are up-regulated individually and may play different pathogenetic roles. IL-1β expression was mainly intrathecal, pointing to its local synthesis within CNS, and although there was a tendency for its lower serum and higher CSF concentrations in more severely ill patients, it could not be definitively confirmed with our data. In WNV-infected mice, IL-1β is expressed independently from TNFα and MIF [26]. Interestingly, in a model studied by Palus et al., higher expression of IL-1β mRNA in the brain tissue was characteristic for the resistant mouse strain suggesting its protective role as opposed to several other pro-inflammatory cytokines, but extending this observation to a human disease requires further study [9].

The expression of TNFα in neurotropic flavivirus infections has been previously described, but its exact role, source, and dynamics is not well defined. The observation of Atrasheuskaya et al. that high serum TNFα concentrations in the first week of hospitalization correlate with a clinically severe TBE [43] and its up-regulation dependent on MIF detected by Arjona et al. in WNV-infected mice [26] prompted us to study its role alongside MIF in initiating the BBB disruption. Interestingly, TNFα may be also involved at other tissue locations and stages of the disease. In mice studied by Hayasaka et al., high TNFα expression in periphery was observed late in the course of TBE, representing the late phase of the excessive systemic inflammation, not directly linked to CNS pathology [7]. On the other hand TBEV induces TNFα synthesis in dendritic cell cultures after 24-h incubation, so its activity may begin already locally in the skin at the tick bite site with the involvement of the resident dendritic cells [44]. Finally, the role of TNFα in the intrathecal response has been suggested by its expression in brains of TBE-infected mice [9, 37]. Intrathecal TNFα expression correlated with fatal outcome in a group of 118 patients with Japanese encephalitis studied by Winter et al. as well [45]. Surprisingly in the light of the above findings, the median TNFα levels in serum and CSF were not significantly increased in our study group, unlike in the much smaller group of patients with clinically milder non-TBE meningitis, suggesting its relatively small role in TBE. This lack of an evident TNFα expression would preclude the presence of a singling pathway analogous to what was described in WNV-infected mice and make it unclear how MIF and TLR3 function could contribute to BBB disruption in the absence of TNFα [26, 33]. However, as discussed below, genotyping data show that TNFα expression in TBE varied in association with the genetic background and that it was in fact up-regulated and could be biologically active in a large subgroup of patients.

The either protective or immunopathogenic role of MIF has been described in several bacterial and viral infections. In the community-acquired pneumonia, a high-expression allele MIF-173C associates with higher IL-6 and IL-10 concentrations and a better prognosis [20]. In the CNS infections however, the trend is the opposite, as a high concentration of MIF in serum and CSF correlates with a more pronounced CNS infection and severe clinical presentation in patients with purulent meningitis [21]. The high concentration of MIF in cerebrospinal fluid (CSF) and presence of high-expression variants 7-CATT and MIF-173C correlate with an unfavorable outcome in *Streptococcus pneumoniae* meningitis, and MIF neutralization is beneficial in the animal model of the disease [22]. The frequency of 7-CATT is also higher in patients with pneumococcal meningitis than in the general population, suggesting that the tendency for a high MIF expression is not only unfavorable during infection, but also associated with an increased risk of its development [23, 24]. Two studies investigated but did not observe evidently increased MIF expression in patients with viral meningitis, although in the study by Østergaard et al., MIF concentration in CSF correlated with its inflammatory parameters [21, 24]. However, MIF is up-regulated in serum and correlates with a severe clinical presentation and a risk of fatal outcome in dengue, which is a systemic flavivirus infection [25], suggestive of its role in the pathogenesis of flavivirus meningitis/encephalitis as well. In fact, the existing data suggest MIF is up-regulated and plays a role in the pathogenesis of encephalitis caused by flavivirus, which may be characteristic for this particular etiology [21, 24, 26]. In Japanese encephalitis virus (JEV) infection in mice, MIF is synthesized in neurons and glial cells prior to TNFα and triggers intrathecal inflammation with resulting tissue damage [27]. In WNV encephalitis model studied by Arjona et al., MIF appears crucial for the CNS invasion, while in patients with WNV encephalitis, MIF serum concentrations were increased, the median concentration in CSF was an order of magnitude higher than in serum and a very high expression was confirmed in the brain parenchyma of two patients who died [26]. In our study, MIF was up-regulated in serum to higher concentrations than in CSF early in the neurologic phase of TBE, consistent with its implied role of a mediator of the systemic inflammation. Serum MIF concentrations tended to correlate with AQ, suggesting its link to BBB disruption. The correlation was statistically significant only in meningitis patients and after exclusion of one case, while the meningoencephalitis group was highly heterogeneous with respect to both parameters, possibly reflecting different timing of the sample collection in relation to the onset of encephalitis or the individual variability. The similarly increased serum MIF concentration in patients with non-TBE meningitis did not correlate with AQ. Although this group was relatively small, the lack of any positive trend suggests that the tendency could, if confirmed, be indeed specific for TBE, or, more generally, for neuroinfections of flavivirus etiology, and that it might reflect the mechanism of BBB impairment characteristic for them.

TLR3 is supposed to play an important protective role during CNS viral infections. It is expressed constitutively by a subpopulation of human neurons [46] and in a study by Farina *et al.*, it was virtually the only functional TLR on human astrocytes, suggesting its importance in the intrathecal innate immunity [47]. The crucial role of TLR3 in the response to herpes simplex type 1 encephalitis has been suggested by Zhang et al. [48]. On the other hand, as TLR3 receptor seems involved in BBB disruption in the mouse model of WNV encephalitis, its effect in flavivirus neuroinfections could be paradoxically detrimental [33]. Kindberg et al. have studied two common SNPs in *TLR3* gene, rs3775291 and rs5743305, in a group of 128 patients with TBE, 77 with viral meningitis/meningoencephalitis of other etiology, and 138 healthy persons from Lithuania. The homozygocity for the low-expression mutant T allele in rs3775291 was found in 19% of the controls and 21% of the patients with non-TBE neuroinfection but only in 7% of the TBE patients [39]. Similarly, Mickienė et al. detected the low-expression rs3775291 TT genotype almost twice less frequently in TBE patients than in the healthy population. However, if present in TBE, the T allele and TT genotype were associated with a more severe clinical presentation [38]. This apparent paradox could be explained by the hypothesis that TLR3 expression indeed facilitates the onset of the neurologic disease by supporting the TBEV penetration through BBB, but has a protective effect during the established CNS infection. Barkhash et al. have confirmed the protective role of the T allele in patients from Novosibirsk (Russia) [40]. The homozygocity for the wild type allele was present in 53.3% of cases of symptomatic TBEV infection and in 62.5% of cases of encephalitis or myelitis, so higher TLR3 expression was associated with a more severe manifestation [40]. This difference between the studies by Mickiene et al. and Barkhash et al. has not been explained, but it could be caused either by a difference between TBEV subtypes and strains prevalent in the locations of both studies or by a different genetic background in the studied populations.

Our study did not confirm correlation between rs3775291 genotypes and TBE frequency or severity, possibly because the low-expression allele was less frequent than previously described, on the level of 7% TT homozygocity in the healthy controls. On the other hand, the frequency of TT homozygocity in rs5743305 locus was higher in TBE group than in controls, although in a study by Kindberg et al., this particular SNP did not associate with TBE risk or severity [39]. This SNP, less studied than rs3775291, is located in the promoter region of *TLR3* and supposed to affect its transcriptional activity. In a study by Dhiman et al., the AT heterozygocity in this locus correlated with a weaker serologic response to measles vaccination, confirming the association of rs5743305 with anti-viral response [49]. Interestingly, the protein concentration in the convalescent CSF was higher in the bearers of TT and TC genotypes in rs3775291 and of the TC/AT combined rs3775291/ rs5743305 genotype. The difference was present only in the convalescent CSF and not directly related to the BBB function in the early phase of the disease. The higher protein concentration was associated with allele either previously described as protective (T in rs3775291) or found to be protective in our study (A in rs5743305). As the low-expression variants protective in the context of early BBB permeability should also associate with a weaker intrathecal response during neuroinvasive disease, the observed trend may represent a less effective control of the CNS infection resulting in a delayed resolution of the inflammation.

The data on rs755622 are to our knowledge the first on *MIF* variability in TBE. The trend in the distribution of the high-expression C allele and CC genotype was statistically weak because of their low frequency, but consistent with our hypothesis. There were only three subjects with a high-expression CC genotype, all belonging to TBE group and presenting with uncomplicated meningitis. Such a distribution is analogous to findings of Mickiene et al. regarding *TLR3* polymorphism and consistent with MIF expression not only facilitating the onset of the neurologic disease but also playing a protective role during the CNS infection. Interestingly, two meningitis patients with CC genotype had atypical composition of the CSF cellular infiltrate, with very low total and lymphocytic pleocytosis suggestive of a peculiar pathology. As the CG genotype was relatively frequent, but did not associate with TBE risk and presentation, the heterozygocity in rs755622 seems not to have a significant influence on the response to TBEV.

Finally, we have analyzed the association of the expression of the studied cytokines with SNPs in *MIF* and *TLR3*, although the strength of the analysis was limited by a relatively small size of the cytokine study group. Unfortunately, no CC homozygotes in *MIF* rs755622 were detected in this group so it remains to be determined to what degree this genotype actually influences MIF expression in TBE. However, there was an expected trend for lower cytokine concentrations associating with the protective *TLR3* rs5743305 A allele, close to reaching the statistical significance for TNFα. Of note, TBE patients with rs5743305 TT genotype had significantly up-regulated serum TNFα levels, while in AT genotype, the increase was non-significant and in AA the serum TNFα expression was hardly detectable. As TT was identified as a high-TBE-risk genotype, this result agrees well with TNFα expression promoting the disease onset and provides a link between the TLR3 expression and the TNFα synthesis during an infection with TBEV in vivo. Even more importantly, the variability associated with rs5743305 explains the unexpectedly low median TNFα concentration in a TBE group and proves that the expression of this cytokine may play a pathogenetic role in a large subgroup of patients. The data show that the suspected pathway involving TLR3, MIF, and TNFα may be functional in human TBE, but that its expression should be highly variable individually in association with the genetic background, which in turn should contribute to a variability of clinical presentation.

## Conclusions

Several of our findings support the hypothesis that the signaling route involving TLR3, MIF, and TNFα is activated during the TBEV infection, participates in the pathogenesis of TBE, and may contribute to the BBB dysfunction. These include increased peripheral concentration of MIF, increased TNFα concentration only in patients with a high-expression *TLR3* genotype and, if confirmed, the tendency for the correlation of the serum MIF level with CSF AQ. The associations of SNPs in *TLR3* and *MIF* with the neuroinvasive TBEV infection, of *TLR3* with the prolonged intrathecal inflammation, and of *MIF* rs755622 with the clinical presentation are consistent with the TLR3- and MIF-dependent activity increasing the risk of the progression to a clinically overt CNS involvement while in the same time decreasing its severity. This would suggest it to be pathogenic and detrimental during the peripheral and/or at the onset of the neurologic phase but protective in the intrathecal infection.

If activated already in the peripheral phase of the infection, MIF signaling route could favor TBEV invasion into CNS by initiating an early increase in BBB permeability, in analogy with the animal models of WNV encephalitis [26, 33]. This mechanism remains speculative, especially that other animal studies suggest that BBB disruption in TBE occurs only after the onset of encephalitis, driven by intrathecal factors [37], and could not contribute to CNS infection. The direct assessment of the BBB function and MIF expression in the peripheral phase of TBEV infection would be necessary to clarify this issue but is difficult to perform in human infection in a clinical setting.

Our data suggest that different genotypes within *TLR3* and *MIF*, and possibly also within genes coding other elements of the same signaling cascade, including receptors for MIF and TNFα, could correspond to either a higher risk of the CNS infection by TBEV with a relatively mild presentation or to a less probable but more severe CNS involvement. This would mean the genetic background may influence the course of the TBE in a complex, multi-step, and sometimes paradoxical manner, contributing to its significant clinical variability and unpredictability. If confirmed, it would be important to investigate to what degree these observations may be applied to the pathogenesis of infections with other neurotropic *Flavivirus* species, including WNV.

## Abbreviations

AQ: Albumin quotient
BBB: Blood-brain barrier
CNS: Central nervous system
CSF: Cerebrospinal fluid
IFN: Interferon
IL: Interleukin
JE: Japanese encephalitis
JEV: Japanese encephalitis virus
M: Meningitis
ME: Meningoencephalitis
MEM: Meningoencephalomyelitis
MIF: Macrophage migration inhibitory factor
SNP: Single nucleotide polymorphism
TBE: Tick-borne encephalitis
TBEV: Tick-borne encephalitis virus
TLR: Toll-like receptor
TNFα: Tumor necrosis factor alpha
WHE: Hardy-Weinberg equilibrium
WNV: West Nile virus
